# Cost-effectiveness analysis for implementation of smoking cessation strategies at primary health care settings in Tamil Nadu

**DOI:** 10.1371/journal.pone.0318013

**Published:** 2025-01-29

**Authors:** Vasantha Mahalingam, Ramesh Kumar Santhanakrishnan, Muniyandi Malaisamy, Karthick Chelvanayagam, Kavi Mathiyazhagan, Adhin Bhaskar, Karikalan Nagarajan, Jerard Maria Selvam, Surendran Veeraiah, Kavitha Rajsekar, Kirti Tyagi, Ponnuraja Chinnaiyan

**Affiliations:** 1 ICMR-National Institute for Research in Tuberculosis, Chennai, India; 2 National Health Mission, Ministry of Health and Family Welfare, Government of Tamil Nadu, Chennai, India; 3 Department of Psycho-Oncology & RCTC, Cancer Institute (WIA), Chennai, India; 4 Department of Health Research, Ministry of Health and Family Welfare, New Delhi, India; Institute for Social and Economic Change, INDIA

## Abstract

**Background:**

Smoking is a major public health concern in Tamil Nadu, as it is in many parts of the world. It is a leading cause of preventable diseases and deaths, with a significant economic burden on healthcare systems and society as a whole. Recognizing the need to address this issue, the implementation of smoking cessation strategies at primary health care (PHC) settings has gained attention. Conducting a cost-effectiveness analysis in this context can help policymakers and healthcare providers make informed decisions about the allocation of resources for such interventions.

**Objectives:**

To compare the cost-effectiveness of the smoking cessation of proposed strategies (PSs), PS1: enhanced counselling (EC) + nicotine replacement therapy (NRT) + bupropion tablet; PS2: behavioural intervention (BI) + NRT + promotion of bupropion sustained release (SR); PS3: EC + NRT + promotion of bupropion SR with the current strategy (BI +NRT+ Bupropion) in a population of smokers aged ≥15 years attending the PHC in Tamil Nadu.

**Methods:**

In this hypothetical cohort of 100,000 individuals using the decision tree analysis, a cost-effectiveness assessment was conducted for both proposed and existing strategies. The results were evaluated in terms of incremental cost-effectiveness ratios (ICERs) per person quitting smoking. To assess the robustness of the findings, one-way sensitivity analysis and probabilistic sensitivity analysis were performed which aims to explore and address the uncertainties associated with the outcomes.

**Results:**

The cost of the current strategy (CS) was higher (₹359 or $4.28 million) when compared with PS1 (₹327 or $3.90 million) and PS3 (₹327 or $3.90 million) strategies. The PS2 with BI + bupropion SR + NRT was found to be more cost (₹2,720,571 or $ 32,414.76) as compared to current strategy. ICER values indicates that compared to the current strategy, the PS1 and PS3 were found to be cost-saving, whereas the PS2 was found to be cost-effective. The cost-effectiveness acceptability curve demonstrated that the PS1 and PS3 indicates 100% probability of the intervention being cost-saving. After excluding dominated interventions (PS2 and CS), the remaining strategies (PS1 and PS3) were compared. The PS3, with an incremental cost of ₹462,497 ($5,510) for 131 additional quitters, resulted in an ICER of ₹3,531 ($42) per quitter, making it a cost-effective option compared to PS1.

**Conclusion:**

Our study findings indicate that the need for healthcare providers and policymakers to implement PS3 with EC, NRT, Bupropion SR, as which was found to be cost-saving compared to current practices.

## 1. Introduction

Every year, tobacco usage takes the lives of more than eight million individuals and is currently the single leading preventable cause of deaths globally [1]. According to World Health Organisation (WHO) estimates, tobacco usage resulted in 100 million preventable deaths globally throughout the 20^th^ century. It was predicted that if the current trend in tobacco use continue, this number would increase to one billion in the 21^st^ century [2]. With 267 million tobacco smokers living in the country, India is the world’s second biggest tobacco consumer [3]. There are wide variations across various states of India in the overall prevalence of tobacco use as well as different forms of tobacco use such as smoking and chewing [4]. Bidi, Cigarette and Hookah are few forms of smoking tobacco. It was reported that the prevalence of usage of tobacco in whatever form among adults was 29%, tobacco smoking was 11% and tobacco chewing was 21% [5]. The main forms of tobacco consumed in India were 11% by *Khaini* (Chewing) and 8% *Bidis* (Smoking). In Tamil Nadu, as per National Family Health Survey (NFHS-5), 20.1% of men aged 15 years and above use any kind of tobacco compared to 4.9% of women in the same age group underscoring a notable gender gap in tobacco use [6]. According to Global Adult Tobacco Survey (GATS-2), 21.1% of men, 0.1% of women and 10.5% of all adults currently smoke tobacco in Tamil Nadu [5].

One of the main risk factors for cancer, heart disease, diabetes, stroke, chronic lung disease, infertility, blindness, TB and oral cavity infections is tobacco smoking. It was reported that 50% of cancers in males and 20% of cancers in females are due to tobacco use. The economic costs related to tobacco use among persons aged 35–69 is ₹1,045,000 million ($124.61 billion) in India [7]. In 2007–08, Indian Government introduced the National Tobacco Control Programme (NTCP) to create awareness, minimise tobacco usage and implementation of ‘The Cigarettes and Other Tobacco Products Act’ (COTPA) was done after recognizing the significance of addressing the tobacco-related problems. The COTPA was enacted in 2003 and forbade the sale of tobacco in any form to minors, smoking in public places and smoking nearer to the educational institutions. It is comprised of the mandatory display of pictorial warnings and testing of tar and nicotine content of all tobacco products [8]. Further the Ministry of Health and Family Welfare (MoHFW), Government of India, with the help of WHO started Tobacco Cessation Clinics (TCCs) to provide cessation strategies including pharmacological treatment for all forms of tobacco users. They also provide toll free telephonic counselling by expert counsellors in all languages and also provide smoking cessation advice through mobile text message. Primary Health Centre (PHC) is the most accessible facility for getting advice and support for tobacco cessation.

ICMR-National Institute for Research in Tuberculosis (NIRT) conducted a cluster randomised controlled trial to compare the effectiveness of smoking cessation strategies among tuberculosis (TB) patients treated at National Tuberculosis Elimination Programme (NTEP) centres in Kancheepuram and Villupuram districts of Tamil Nadu [9]. The study compared three interventions for smoking cessation among TB patients, namely (1) Bupropion Sustained Release (SR) 150mg for 7 weeks daily with standard counselling, (2) Enhanced Counselling (EC), (3) Standard counselling (SC) or behavioural intervention (BI). The EC was a motivational package including a) Brochures on smoking cessation provision b) Flip charts c) Posters d) movie and e) family counselling. This package will provide information on the hazards of smoking, ways to quit smoking, withdrawal symptoms and family benefits on quitting smoking. The BI recommends providing brief advice to quit smoking, including the 5 ‘A’ approach (Ask, Advise, Assess, Assist and Arrange) and the 5 ‘R’ approach (Relevance, Risk, Rewards, Roadblocks and Repetition) [9]. Bupropion is non-nicotine drug and used to treat tobacco dependency. It is an atypical antidepressant with adrenergic and dopaminergic effects. Treatment for major depression disorder with bupropion SR provides a sustained-release approach. Since it strikes a compromise between effectiveness and tolerance, bupropion SR is frequently chosen because it lowers the risk of adverse effects and streamlines the dosage schedule.

In the study, the trained NTEP health care workers delivered the various cessation strategies and assessed smoking status after 2-months from the initiation of anti-TB treatment and at the end of treatment. The percentage of patients who quit smoking at the end of TB treatment in three arms of SC, drug with SC and EC were 52%, 67% and 83% respectively. It was also observed that favourable TB outcome was higher among those who quit smoking. The study findings emphasized the needs of including EC for tobacco cessation in lieu of ‘standard of care’ for effective management of tobacco cessation under NTEP. Based on these results, the Government is planning to implement this strategy not only for TB patients under NTEP but also to all smokers under NTCP. The EC can also be extended to NTCP in addition to NTEP as an effective smoking cession intervention. In line with this, we have undertaken this health technology assessment (HTA) study along with the Government of Tamil Nadu to evaluate the cost-effectiveness of comprehensive approach of smoking cessation that combines both BIs and pharmacological support implementation at PHCs in Tamil Nadu.

## 2. Methods

We modelled the cost-effectiveness of smoking cessation strategies by a decision tree method from a health system perspective. The current study assessed the cost-effectiveness of three proposed strategies (PSs) and the current strategy (CS) for smoking cessation and in a population of smokers aged ≥15 years attending the PHCs in Tamil Nadu.

### 2.1 Settings

India is one among the 29 nations worldwide that have entirely outlawed tobacco sponsorship, advertising and promotion. In order to offer tobacco cessation interventions, the MoHFW, Government of India, established TCCs across the country with assistance from the WHO and currently there are 427 TCCs under NTCP. In addition, there are 179 non-NTCP tobacco cessation facilities working in the country. These facilities were set up in different settings such as medical colleges, Non-Governmental Organisations, community settings, cancer treatment hospitals and psychiatric hospitals to help the users to quit tobacco use. Over 600 districts nationwide are currently covered by the NTCP, which is being executed [10]. NTCP is executed through a three level system which are National Tobacco Control Cell (NTCC), State Tobacco Control Cell (STCC) and District Tobacco Control Cell (DTCC).

### 2.2 Study design

This study focused on the implementation of smoking cessation strategies at PHC settings in Tamil Nadu among the population of smokers of age more than or equal to 15 years. Previously, ICMR-NIRT conducted a cluster randomised trial on smoking cessation for TB patients. The trial result showed that EC and pharmacological therapy are effective strategies for smoking cessation. Based on the findings, this current study is assessing cost-effectiveness of three combinations of smoking cessation strategies compared to the CS using a decision-analytic method. In this cost-effectiveness analysis, the population of smokers are divided based on severity of the nicotine addiction using the Fagerström index (FI) score [11, 12] and the treatment is given according to their FI score. We also aimed to calculate the additional costs related with the proposed strategies as the interventions from the health system perspective.

### 2.3 Study population

The study is conducted taking into consideration of general population of smokers aged ≥15 years who visits PHC. The population is divided according to their FI score as 0–2 (low dependence), 3–5 (low to moderate dependence), 6–7 (moderate dependence) and ≥8 (high dependence) [11]. Study population represents a hypothetical cohort of 100,000 smokers aged ≥15 years.

### 2.4 Study perspective

A health system perspective was used for this cost-effectiveness evaluation which considered only the costs incurred by the health system such as the cost for doctor, social/health care worker, drug costs for nicotine replacement therapy (NRT) 2mg, NRT 4mg, Bupropion, Bupropion SR, costs for Information Education and Communication (IEC) materials preparation, Digital Versatile Disc (DVD) and flip chart.

### 2.5 Intervention and comparator

The interventions or PSs considered are the three combinations of BI, EC and the drugs such as bupropion SR, NRT 2mg, NRT 4mg. The PS1 involves EC + NRT + Bupropion tablet. The PS2 strategy involves BI + NRT + Promotion of Bupropion SR. The PS3 involves EC + NRT + Promotion of Bupropion SR (Table 1). The CS used for smoking cessation is considered as the comparator. The CS involves BI + NRT+ Bupropion. The dosage of NRT increases as the FI score increases.

**Table 1:** Strategies for smoking cessation.

Strategies	Level of implementation	Smoking cessation steps
Proposed Strategy-1	Primary Health Care Centres	EC: Motivational Package includes Brochures, Flip charts, Posters, Movie/video presentations, Family counselling + NRT (nicotine patches, gums, spray, inhaler, sublingual tablets and lozenges) + Bupropion
Proposed Strategy-2		BI + NRT + Promotion of Bupropion SR
Proposed Strategy-3		EC + NRT + Promotion of Bupropion SR
Current Strategy		BI + NRT + Bupropion

BI = Behavioral Intervention; EC = Enhanced counselling; SR = Sustained Release; NRT = Nicotine Replacement Therapy

### 2.6 Model description

**Fig 1:**
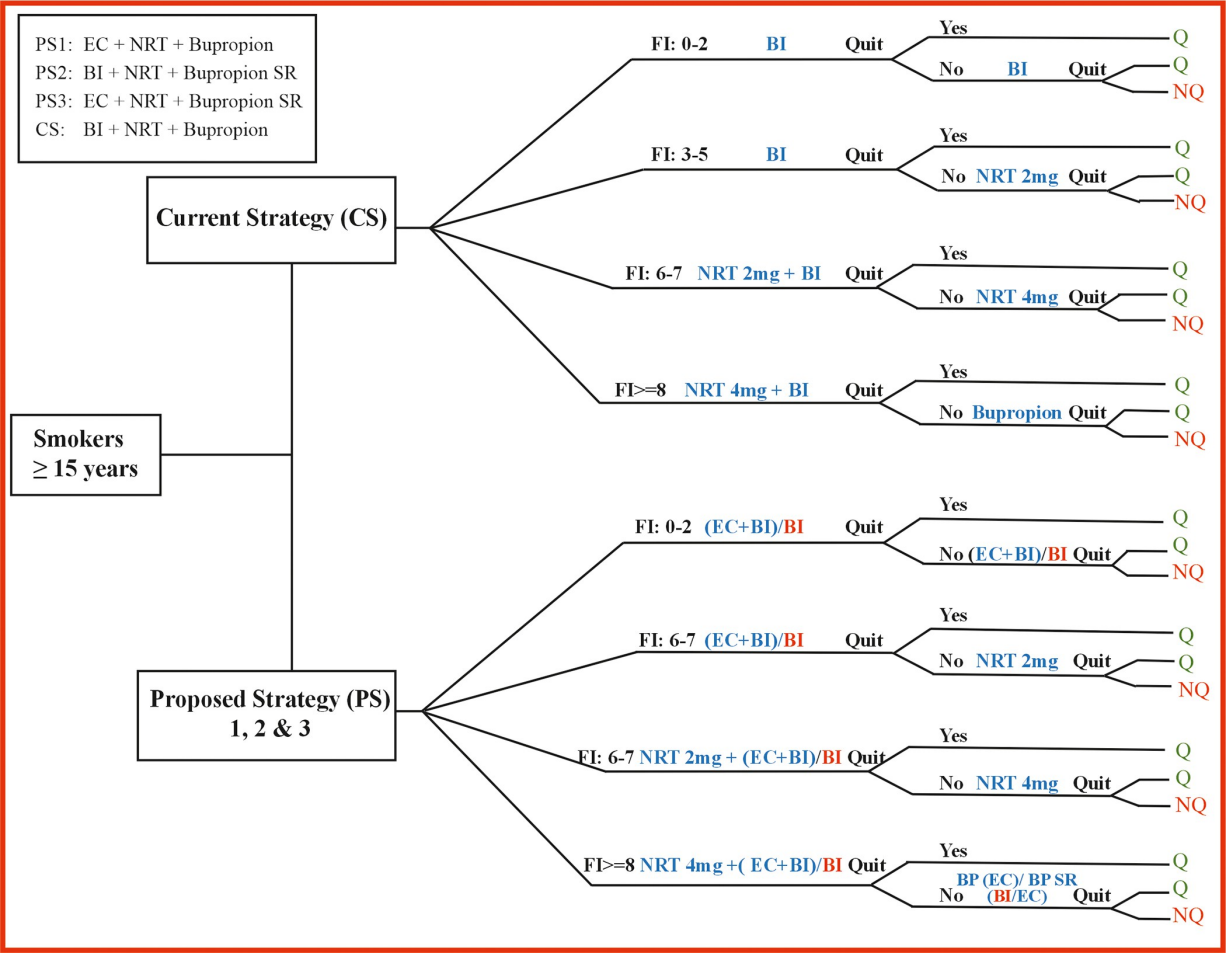
Decision tree. BI = Behavioral Intervention; EC = Enhanced counselling; SR = Sustained Release; NRT = Nicotine Replacement Therapy; FI = Fagerstorm Index (FI); Q = Quit; NQ = Not Quit.

The deterministic decision tree model for cost-effectiveness analysis was performed in Microsoft Excel. The decision tree is given in Fig 1. Three PSs were investigated for the analysis, PS1: EC + NRT+ Bupropion; PS2: BI + NRT+ Bupropion SR and PS3: EC + NRT + Bupropion SR. The input data for the model were obtained from various sources such as published articles from the secondary sources. The model had been calibrated reflecting the population characteristics and examined in a hypothetical cohort of 100,000.

### 2.7 Decision tree

The decision tree for assessing smoking cessation probability categorizes individuals into four branches based on their FI scores: 0–2, 3–5, 6–7, and ≥8, representing low, low to moderate, moderate and high dependence, respectively. Within this framework, in the CS receives BI +NRT+ Bupropion as part of the existing strategy. The proposed intervention arm introduces three strategies, each involving modifications to the CS by additionally incorporating EC and bupropion SR. PS1integrates EC alongside bupropion, expanding the decision tree into four branches for individuals falling within the ranges of FI scores 0–2, 3–5, 6–7, and ≥8. PS2 implements BI combined with bupropion SR, similarly extending the decision tree across these four FI-based branches. PS3 is same as PS1 but pairs EC with bupropion SR, thus reforming the intervention pathways within the decision tree across different FI score categories. These PSs aim to diversify and enhance smoking cessation interventions accommodating various FI scores.

### 2.8 Model input parameters

The input parameters were cohort population, mean age of smokers, prevalence of smokers according to FI score and probability of quitting smoking by respective treatment strategies. The input parameters are given in the Table 2. The cost acquired by health system in regard to the implementation of the smoking cessation were included in the model. The data were collected from the published literature.

**Table 2:** Input parameter used in the cost effectiveness analysis for implementation of smoking cessation strategies.

Variable		Base case	Lower	Upper	Distribution	Source
**Demographic**	Average age of smokers	18.2	13.65	22.75	Normal	^3^
	Cohort population	100,000	-	-	NA	^a^
**Prevalence**	Smokers with FI:0–2	0.02	0.015	0.025	Beta	^7^
	Smokers with FI:3–5	0.11	0.083	0.138	Beta	^7^
	Smokers with FI:6–7	0.62	0.465	0.775	Beta	^7^
	Smokers with FI:≥8	0.23	0.173	0.288	Beta	^7^
**Quit rate**	BI	0.52	0.39	0.65	Beta	^8^
	NRT 2mg	0.6	0.45	0.75	Beta	^11^
	NRT 4mg	0.6	0.45	0.75	Beta	^11^
	Bupropion	0.5	0.375	0.625	Beta	^8^
	Bupropion SR	0.67	0.503	0.838	Beta	^8^
	EC	0.83	0.623	1	Beta	^8^
**Combined effect of quit rate**	EC+BI	0.918	0.689	1	Beta	^b^
	NRT 2mg + BI	0.808	0.606	1	Beta	^b^
	NRT 4mg + BI	0.808	0.606	1	Beta	^b^
	NRT 2mg + EC+BI	0.967	0.725	1	Beta	^b^
	NRT 4mg + EC+ BI	0.967	0.725	1	Beta	^b^
**BI cost**	Doctor cost	₹150 ($1.79)	112.5	187.5	Gamma	^C^
	Social/Health Care Worker	₹76 ($0.91)	57	95	Gamma	^9^
**EC cost**	Doctor cost	₹150 ($1.79)	112.5	187.5	Gamma	^c^
	Social/Health Care Worker	₹76 ($0.91)	57	95	Gamma	^9^
	IEC material cost	₹196 ($2.34)	147	245	Gamma	^8^
**Drug cost**	NRT 2mg	₹3,024 ($36.03)	2,268	3,780	Gamma	^10^
	NRT 4mg	₹3,528 ($42.04)	2,646	4,410	Gamma	^10^
	Bupropion 150mg	₹840 ($10.01)	630	1,050	Gamma	^10^
	Bupropion SR 100mg	₹1,444 ($17.21)	1,082.81	1,804.69	Gamma	^10^
**WTP**	Willingness to pay threshold (GDP per capita) (in INR)	₹235,730 ($2808.65)	-	-	NA	^11^

^a^ = Assumption; ^b^ = Estimated; ^c^ = Expert Opinion; BI = Behavioral Intervention; EC = Enhanced counselling; SR = Sustained Release; mg = milli gram; NRT = Nicotine Replacement Therapy; NA = Not Applicable

### 2.9 Effectiveness data

The proportion of smokers in the FI score classifications were taken from a study on nicotine dependence among smokers in Tamil Nadu [11]. The prevalence of smokers was taken from the GATS survey 2017 [3]. The proportion of smokers receiving BI and NRT/Bupropion was collected from the Tamil Nadu Tobacco survey conducted during 2016–2017. Quit rate of smoking by different interventions such as BI, NRT and EC were taken from the cluster randomized trial conducted in Tamil Nadu [9].

### 2.10 Cost data

The cost data encompassed comprehensive components required for BI, EC such as IEC materials, drugs and various other associated costs. These were collected from a cluster randomized trial conducted by ICMR-NIRT on smoking cessation in TB [9]. These expenses covered various aspects of implementing smoking cessation strategies, including staff cost for counselling [13], IEC material expenses, DVD and flip chart cost. The drug cost for NRT 2mg, NRT 4mg and bupropion were taken from various published literatures [14]. All costs in Indian Rupees (₹) are converted into US Dollars ($) using the exchange rate of 1$ = 83.93₹.

### 2.11 Model outcome parameters

The model’s outcomes were presented in terms of the total number of patients who successfully stopped smoking and the total expenses incurred by each patient in the intervention and comparator arms. Additionally, the ICER (Incremental Cost-Effectiveness Ratio) values were estimated by evaluating the incremental cost and incremental quit rate.

### 2.12 Incremental cost-effectiveness ratio (ICER)

The ICER is used to compare the cost-effectiveness of healthcare interventions. It is calculated by dividing the difference in costs between two interventions by the difference in their effectiveness. Dominated interventions were excluded from the analysis and ICERs were recalculated incrementally for the remaining non-dominated interventions. This ensured that both simple and extended dominance were addressed, resulting in a clearer cost-effectiveness comparison. When ICERs fall below the defined threshold, then interventions are considered to be cost-effective. ICER helps to determine whether the additional benefits of a new intervention justify its additional costs, guiding healthcare resource allocation and ensuring value for money in healthcare decisions.

### 2.13 Willingness to pay

The Willingness to Pay (WTP) criterion for the year 2023 was the one-time GDP per capita of ₹216,590 ($2,580). ICERs were used to compare the cost-effectiveness of the proposed interventions [15].

### 2.14 Sensitivity analysis

One Way Sensitivity Analysis (OWSA) was performed to check robustness of the model by changing the values of input parameters 20% below and above from the normal value. The effect of outcome variable on ICER and the uncertainty in the variables were illustrated in the Tornado diagram. The model validation was done using Probabilistic Sensitivity Analysis (PSA) by 1000 iterations of Monte Carlo simulations with 95% confidential intervals. The resulted values of ICER were plotted using a scatter plot. To indicate the model’s probabilistic response to different cost-effectiveness thresholds, the graph of Cost Effectiveness Acceptability Curve (CEAC) was plotted.

### 2.15 Study oversight

The study used only secondary data from the published literature. All data used and generated in this study are available in the manuscript. The study was approved by Institutional Ethics Committee (ICMR-NIRT IEC No. 2022 028). We adhered good reporting practices from the standard guidelines for conducting and reporting an economic evaluation survey (CHEERS) statement for this study.

## 3. Results

### 3.1 Base case analysis

The base case analysis for hypothetical 100,000 cohort resulted the total costs acquired using different strategies (1) EC + NRT + Bupropion (PS1), (2) BI + NRT + Bupropion SR (PS2) and (3) EC + NRT + Bupropion SR (PS3) when compared with the current strategy (CS: BI + NRT + Bupropion SR) are ₹327 ($3.90), ₹362 ($4.31), ₹327 ($3.90) and ₹359 ($4.28) million respectively. It was observed that the cost of the CS was higher (₹359 or $4.28 million) when compared with PS1 (₹327 or $3.90 million) and PS3 (₹327 or $3.90 million) strategies (Table 3). Whereas the CS was lesser than the PS2 (₹362 or $4.31 million). Among the proposed strategies, the PS2 with BI + NRT + Bupropion SR was found to be costly (₹2,720,571 or $32,414.76) as compared to CS.

**Table 3:** Incremental cost effectiveness analysis before and after removing dominance.

Strategy	Total		Incremental		ICER
	Cost in INR (USD)	No of Quitters	Cost in INR (USD)	Quitters	Cost in INR (USD) per Quitter
EC + NRT + BP (PS1)	327,083,021 (3,897,093)	98,396	-32,784,767 (-390,620)	8,133	-4,031 (-48)
BI + NRT + BP SR (PS2)	362,588,359 (43,20,128)	91,029	2,720,571 (32,414)	766	3,551 (42)
EC + NRT + BP SR (PS3)	327,545,518 (39,02,603)	98,527	-32,322,270 (-3,85,109)	8,264	-3,911(-46)
BI + NRT + BP(CS)	359,867,788 (42,87,713)	90,263	-	-	-
After removing dominance					
EC + NRT + BP (PS1)	327,083,021 (3,897,093)	98,396	-	-	-
EC + NRT + BP SR (PS3)	327,545,518 (39,02,603)	98,527	462,497 (5,510)	131	3531 (42)

PS = Proposed Strategy; CS = Current Strategy; BI = Behavioral Intervention; EC = Enhanced counselling; SR = Sustained Release; NRT = Nicotine Replacement Therapy

### 3.2 Incremental cost effectiveness ratio (ICER)

The value of ICER was estimated using the incremental cost and incremental quit rates. The ICER values for the strategies PS1, PS2 and PS3 when compared with the current strategy (CS:BI + NRT + Bupropion SR) are ₹-4031 ($-48.03), ₹3551 ($42.31) and ₹-3911 ($-46.60) respectively (Table 3). These ICER values indicate that PS1 was cost saving of ₹4031 ($48.03) and PS3 was also cost saving of ₹3911 ($46.60) per person to quit smoking compared to CS. The strategies PS1 and PS3 were found to be cost-saving, whereas the strategy PS2 was found to be cost effective. For PS2, the additional cost to help one person to quit smoking is ₹3,551 ($42.31). After removing the dominated interventions (PS2 and CS), the remaining dominant strategies (PS1 and PS3) were compared. PS3 demonstrated a modest incremental cost of ₹ 462,497 ($5,510) for an additional 131 quitters, resulting in an ICER of ₹3,531 ($42) per quitter. This suggests that PS3 is a cost-effective option when compared to PS1, offering additional benefits at a reasonable cost.

### 3.3 One way sensitivity analysis (OWSA)

**Fig 2:**
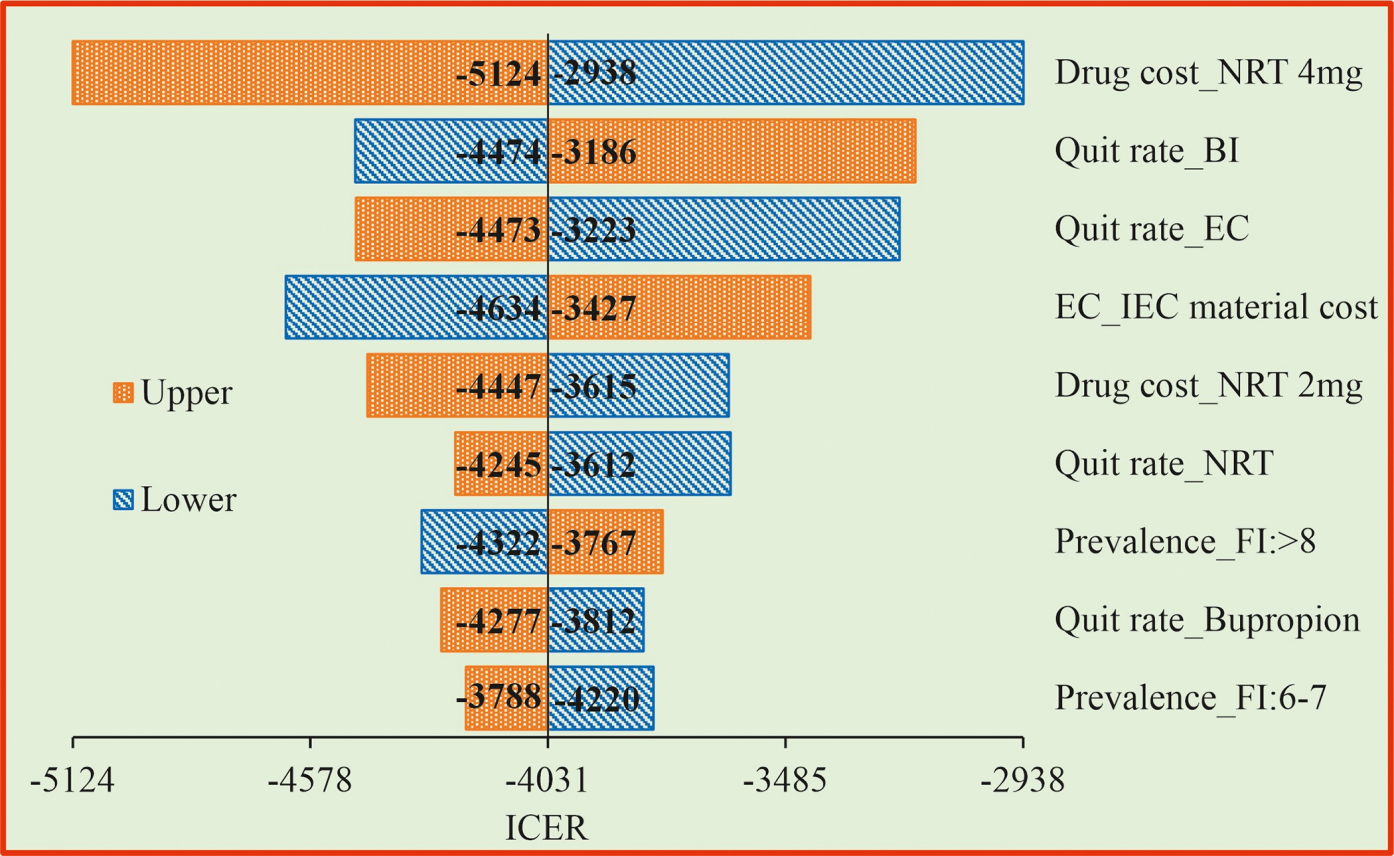
Tornado diagram for EC compared with BI. BI = Behavioral Intervention; EC = Enhanced counselling; NRT = Nicotine Replacement Therapy; FI = Fagerstorm Index (FI); IEC = Information Education and Communication; ICER = Incremental Cost Effective Ratio.

**Fig 3:**
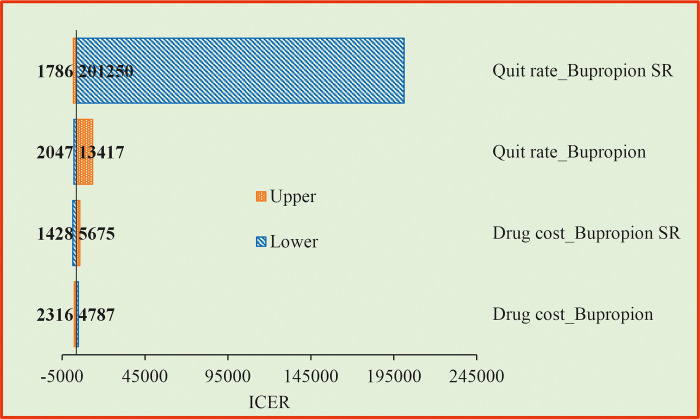
Tornado diagram for promotion of bupropion SR compared with BI. SR = Sustained Release; ICER = Incremental Cost Effective Ratio.

**Fig 4:**
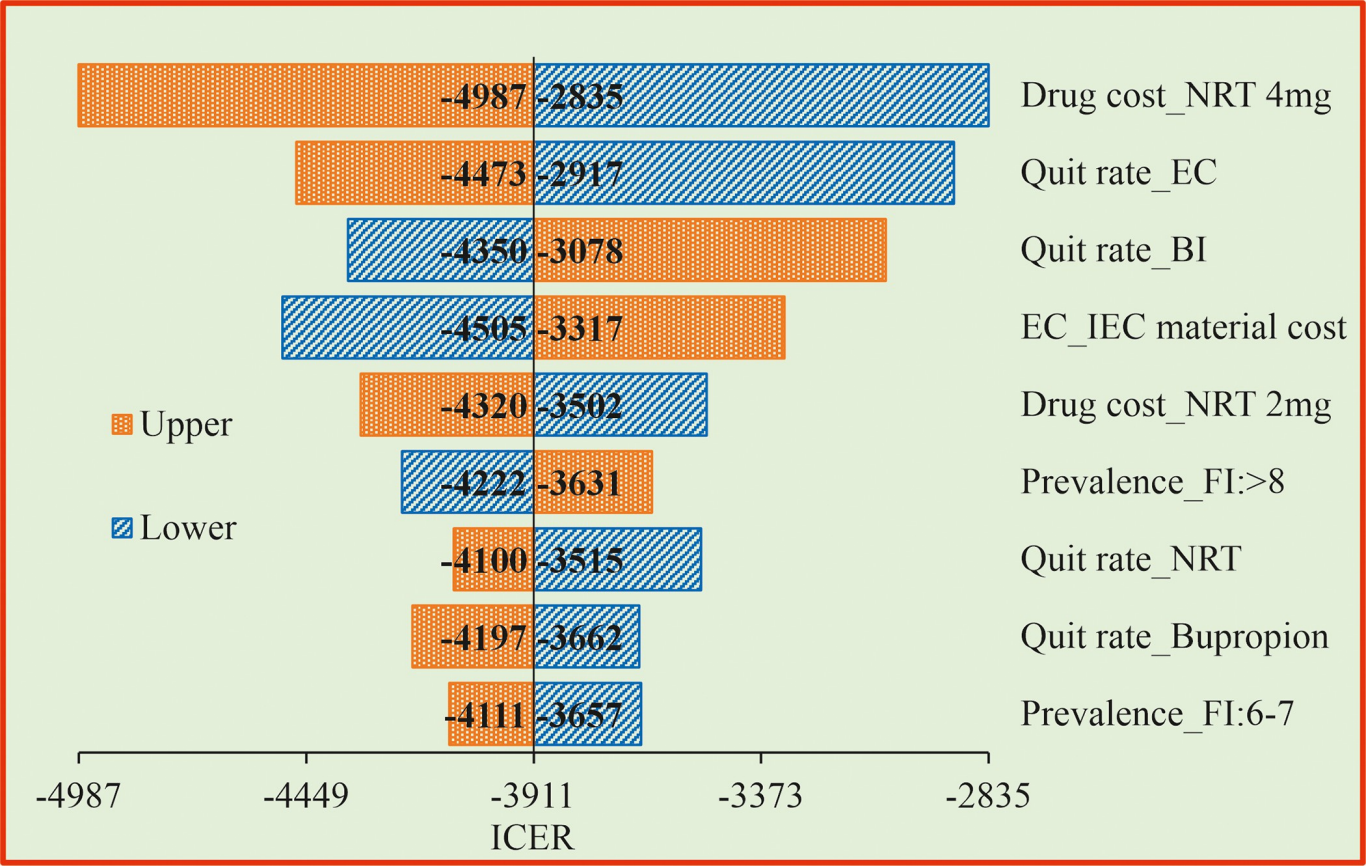
Tornado diagram for enhanced counselling + bupropion SR compared with behavioural intervention. BI = Behavioral Intervention; EC = Enhanced counselling; NRT = Nicotine Replacement Therapy; FI = Fagerstorm Index (FI); IEC = Information Education and Communication; ICER = Incremental Cost Effective Ratio.

The OWSA for the PS1 when compared with the CS revealed that drug cost of NRT 4mg and quit rate by BI had higher influence on the ICER values (Fig 2). When drug cost increases, we need to spend more money to make a person quit from smoking. If the quit rate increases, the ICER value reduces, indicating that we need to spend less amount to get a person quit from smoking. The OWSA for the PS2 when compared with the CS showed that quit rate by Bupropion SR, quit rate by Bupropion, drug cost of Bupropion SR and drug cost of Bupropion are the most influencing factors of the ICER (Fig 3). For the PS3, drug cost of NRT 4 mg, quit rate by EC, quit rate by BI and IEC material preparation cost were found to be the most influencing factors of the ICER when compared with the CS (Fig 4).

### 3.4 Probability sensitivity analysis (PSA)

**Fig 5:**
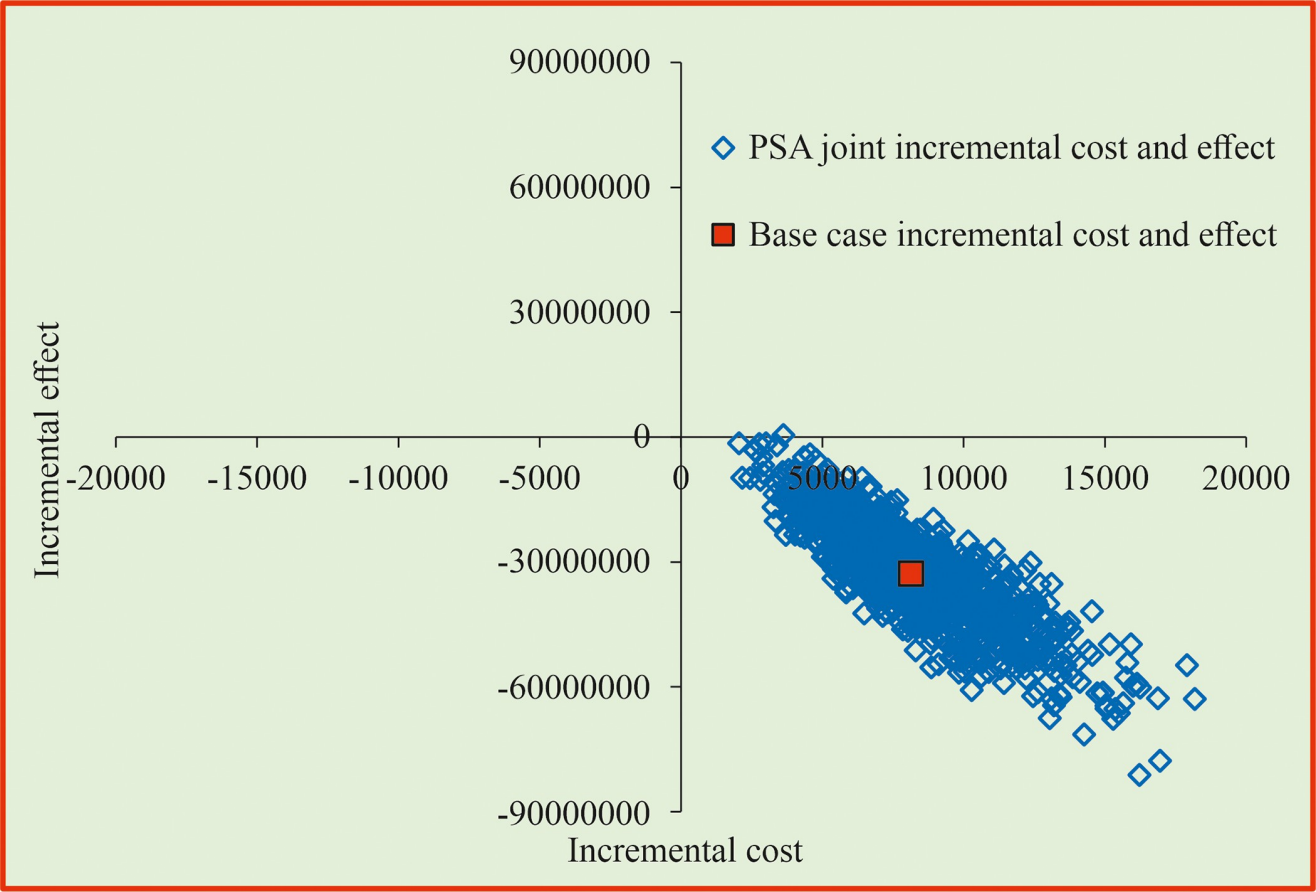
Cost-effectiveness plane for enhanced counselling compared with behavioural intervention. PSA = probability Sensitivity Analysis.

**Fig 6:**
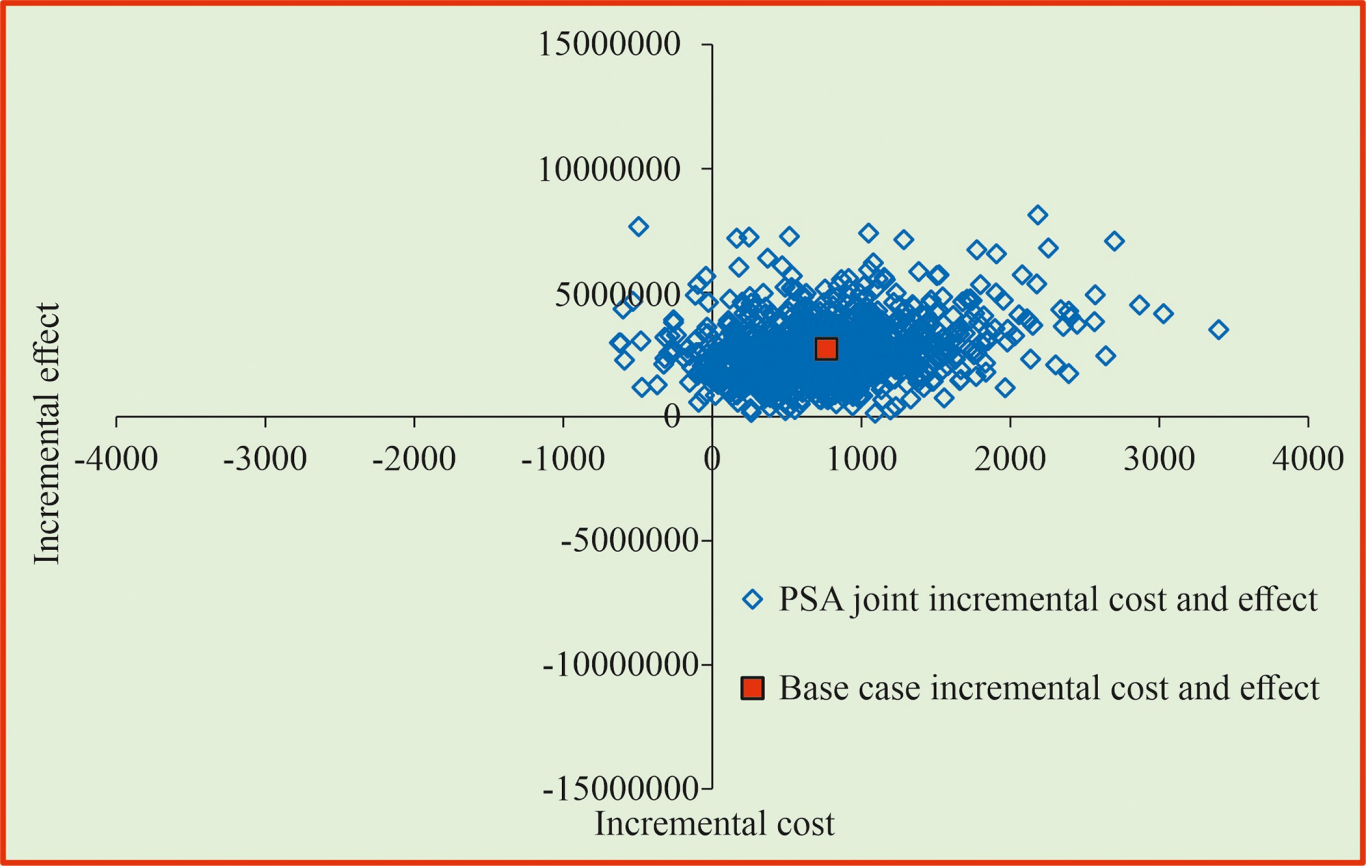
Cost effectiveness plane for bupropion SR compared with behavioural intervention. PSA = probability Sensitivity Analysis.

**Fig 7:**
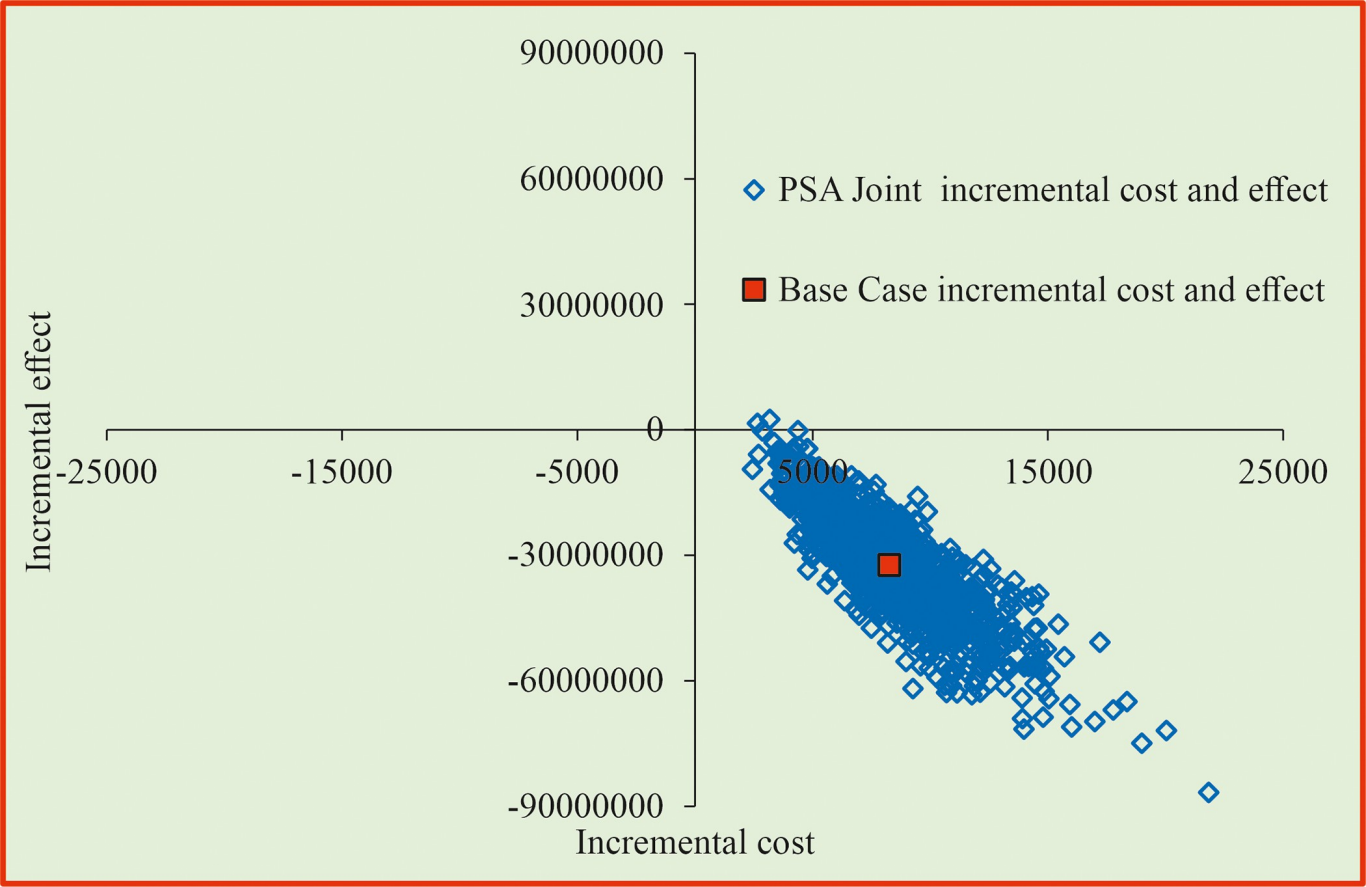
Cost effectiveness plane for enhanced counselling + bupropion SR compared with behavioural intervention. PSA = probability Sensitivity Analysis.

Fig 5 shows that strategy PS1 was found to be less-costly and more-effective in 100% of the iterations using PSA and considering the joint incremental cost and effectiveness measured in quit rate. The PSA results of strategy PS2 indicated that it is more-costly and more-effective in 95% of the iterations (Fig 6). The PSA results of strategy PS3 showed that it is more-effective and less-costly in 100% of the iterations (Fig 7).

### 3.5 Cost-effectiveness acceptability curve (CEAC)

**Fig 8:**
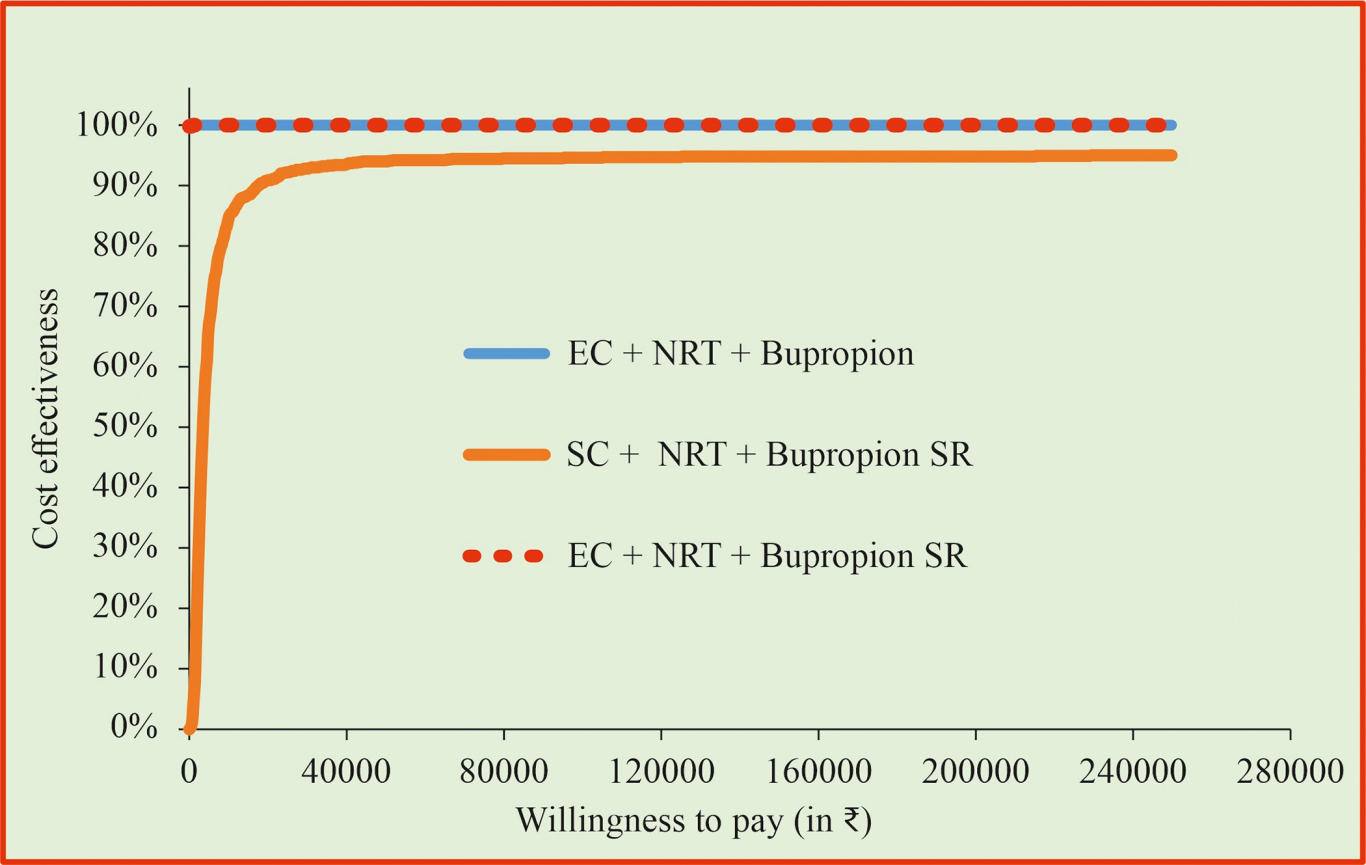
Cost effectiveness acceptability curve. BI = Behavioral Intervention; EC = Enhanced counselling; NRT = Nicotine Replacement Therapy.

The cost-effectiveness acceptability curve demonstrated that PS1 and PS3 indicates 100% probability of the intervention being cost-saving (Fig 8). PS2 indicates that 95% probability of the intervention being cost-effective since none of the ICER values were higher than WTP threshold.

## 4. Discussion

The current study demonstrates that cost-effectiveness of three different smoking cessation intervention strategies as compared to the current practice under the NTCP in Tamil Nadu. The salient finding of this study indicates that the strategy with EC + NRT + Bupropion SR was found to be cost effective as compared to the current practice. Our results support the implementation of smoking cessation with EC, which could yield substantial cost-saving to the health system in Tamil Nadu. It was also evident with the clinical effectiveness report that those who received combined pharmacotherapy and counselling did significantly better than those receiving only counselling [16].

It is often known that quitting smoking is one of the best strategies to save lives and improving overall wellbeing. Quit smoking remains difficult due to the addictiveness of nicotine in tobacco and other social and contextual factors [17, 18]. The behavioural counselling along with pharmacotherapy is generally considered as an effective approach for treating tobacco dependence. Similar finding was reported from a model based economic evaluation on tailored smoking cessation by pharmacological therapy including NRT, bupropion and behavioural interventions that are cost-effective in the normal population [19]. Another study assessed the cost-effectiveness of NRT intervention in primary care setting and found the intervention to be cost-saving ($1065 in savings per person) accounting the decreased health expenses in a lifetime for quit smokers. It was recommended that NRT intervention in primary care setting should be considered in addition to the existing policy to minimise the disease and death related to smoking [20]. Guidelines recommend combining drug therapy and behavioural counselling which help smokers to quit [21, 22]. The current study also supports and re-emphasizes the need for drug therapy and behavioural support to help people quit smoking.

A study on the enhanced smoking cessation for people with severe psychiatric illness showed increased quit rates compared with usual care [23]. A pragmatic randomized controlled trial on enhanced smoking cessation interventions suggested that there is a need to address the unmet need for enhanced smoking cessation in tobacco smoking population. In the study, quit smoking at six months demonstrates that individuals with severe mental illness can successfully stop smoking; nevertheless, the weakening of this effect indicates that continued quitting will require additional work.

It was found from a network meta-analysis study that among COPD patients, amalgamation of behavioural intervention and pharmacotherapy was superior as compared to the effect of varenicline, bupropion and NRT for smoking cessation [24]. Cognitive behavioural therapy combined with bupropion treatment may be the most effective way to help them stop smoking for COPD patients. It was also recommended that in addition to cessation services, close follow-up is crucial [25]. Improving the quality of life for tobacco users involves offering effective smoking cessation interventions.

Smoking cessation is effective in reducing both short and long-term effects of smoking related to morbidity and mortality. As smoking continues to be a major threat for developing heart diseases, cancer and lung disease, it is the need of the hour to implement the most cost-effective smoking cessation strategy in order to reduce the burden of smoking in India [26]. A study from Mumbai had estimated the mortality attributed to tobacco related illness among men and women in the age group of 35–69 years was 24% and 6% respectively [27].

The cost-effectiveness of medication and intensive counselling is often evaluated based on the cost per quality-adjusted life year (QALY) gained for helping the general population quit smoking. Research studies on Nortriptyline, Bupropion and NRT has demonstrated that their cost-effectiveness ratios are consistently less than €10,000 in every (quality-adjusted) life year [28–31]. Studies from other parts of the world conducted on the cost-effectiveness of different strategies for smoking cessation showed that the implementation of an intensive smoking cessation was moderately to highly cost-effective, suggesting that more spending on interventions yields more net benefit [32–35]. Our study findings also highlight that the smoking cessation strategies are less costly and more effective. The most cost-effective strategy is the one which includes EC, NRT and bupropion SR.

The limitations of the current study were that the relapse rate of smokers, non-adherence to treatment and adverse drug reactions were not included as they were not available specific to treatments for smoking cessation. Also, the different factors associated to smoking cessation such as close monitoring, type of communicators, influence of co-morbidities had not been taken into account in this cost and effectiveness data. This might had underestimated the results.

## 5. Conclusion

Compared to the current practices, the implementation of EC, NRT, Bupropion SR and the strategy with EC, NRT, Bupropion were found to be cost-saving. After removing the dominated interventions, the strategy with EC, NRT, Bupropion SR was found to be cost-effective compared to current practices. EC with pharmacotherapy resulted in low costs per quit from smoking. These results re-emphasize the importance of implementing the EC intervention with combining medication and behavioural support to help people stop smoking as it is not only clinically effective but also cost-saving from an economic perspective. Our study findings indicate that the strategy that includes EC and Bupropion SR is the economically dominant strategy to be implemented in the tobacco control programme. Policymakers should consider adopting this strategy to enhance programme effectiveness and cost-efficiency.
